# Laser imaging of ancient tattoos: New tools illuminate established knowledge

**DOI:** 10.1073/pnas.2501049122

**Published:** 2025-03-20

**Authors:** Aaron Deter-Wolf, Benoît Robitaille, Lars Krutak

**Affiliations:** ^a^Tennessee Division of Archaeology, Nashville, TN 37243; ^b^Independent Researcher, Bonsecours, QC J0E 1H0, Canada; ^c^Museum of International Folk Art, Santa Fe, NM 87505

We write in response to the article “Hidden artistic complexity of Peru’s Chancay culture discovered in tattoos by laser-stimulated fluorescence.”(1) Developing technologies for nondestructive archaeological documentation is important, and we thank Kaye et al. for highlighting the utility of laser-stimulated fluorescence (LSF) in recording preserved tattoos. However, we have several concerns with the presentation of this work.

The authors do not include details on their parameters, LSF module, exposure timing, image capture setup, or museum identification numbers. We appreciate the limits of the Brief Report format; nevertheless, this information is critical to independent evaluation of the work and should appear in Supporting Information.

The authors state LSF provides benefits beyond infrared imaging. However, the single infrared photo in the *PNAS* report is out of focus and at lower resolution than the adjacent LSF and natural light images. Prior studies have used infrared and multispectral imaging and digital microscopy to document faint archaeological tattoos at greater detail and clarity than the LSF examples illustrated in *PNAS* (2–5), revealing the precise forensic details of ancient Andean tattoos that Kaye et al. assert have not been previously seen (2, 3).

The final section of the paper employs terminology and presents claims entirely inconsistent with the current state of knowledge on pre-electric tattooing technologies and techniques (6). Kaye et al. incorrectly claim that the documented tattoos are created by puncture using a needle. Prior experimental study and research on remains from the Andes and Europe (2, 4, 7) demonstrate that the specific physical traits of nearly all tattoos illustrated in the *PNAS* article (parallel arrangements of ~0.2 mm wide lines with end tailing and solid edges) are diagnostic of incision tattooing. Conversely, the stippled, variable edges of tattoos in their figure 2H are the result of puncture tattooing. These differences reflect methodological decisions by Chancay tattooists and should not be subject to modern, etic assessments of perceived aesthetic quality.

Most critically, Kaye et al. do not acknowledge work by Peruvian researchers who have described preserved tattoos from the same site (8–10), and previously established the intricacy of Chancay tattooing that they claim to have now “discovered.” One of those earlier reports (9) illustrates the same Chancay vessel presented in figure 1 of the PNAS article. Another, by van Dalen Luna et al. (8), illustrates preserved tattoos composed of short, thin parallel lines identical to those presented in PNAS. The earlier authors correctly identify these, along with the majority of Chancay tattooing at Cerro Colorado, as being done by incision.

The critiques presented here are not based on differences of opinion. Instead, the shortcomings of this article stem from inadequate engagement with key source material. Responsible peer-reviewed science should not present hypotheses in opposition to established knowledge without fully testing those claims. It is imperative that scholars writing about past cultures present information that is accurate and in step with current understandings. Not doing so undermines the efforts of other researchers and descendant communities to comprehend and value the associated cultural heritage.
